# New patients to the Leeds spondyloarthritis clinic: is referral stratification working?

**DOI:** 10.1186/1471-2474-14-S1-A14

**Published:** 2013-02-14

**Authors:** Rebecca C Thomas, Helena Marzo-Ortega

**Affiliations:** 1 Department of Rheumatology, Chapel Allerton Hospital, Leeds LS7 4SA, UK

## Background

Treatment of spondyloarthritis (SpA) has advanced enormously in recent years, with anti-TNF treatment taking a central role. National and international guidelines [1-3] suggest that anti-TNF treatment for ankylosing spondylitis (AS) and psoriatic arthritis (PsA) should be commenced where possible by a rheumatologist with a specialist interest in these conditions. It is therefore important that patients are reviewed by the appropriate clinician where possible, and this is the rationale behind the weekly Leeds SpA clinic.

Patients may be referred by their general practitioner (GP) or community musculoskeletal services. Some are referred from other medical specialties, notably dermatology and gastroenterology. Referrals reach the clinic in two ways: either via the “choose and book” system if directly requested by GP, or by a general referral that is then triaged by one of the six clinical rheumatology trainees in Leeds. The Leeds SpA clinic is presently oversubscribed, so appropriate identification of new patients to this specialist clinic is important. This audit sought to review referrals to this clinic to assess the current efficacy of the referral system.

## Methods

A retrospective audit was performed, with appropriate referral of SpA being the gold standard. New referrals seen in the Leeds SpA clinic over a six month period (June 2011-November 2011 inclusive) were reviewed using electronic letters and electronic results server. Audit approval was obtained from Leeds Teaching Hospitals.

## Results

75 new referrals were identified as having attended a “new patient” appointment. The mean patient age was 41.13 years, with median 42.5 years. 41 of the referred patients were male. 45 patients had a diagnosis of spondyloarthritis, with the majority (n = 24) being diagnosed with PsA. A total of 55 patients had features in the history that could be suggestive of a diagnosis of spondyloarthritis, with a personal history of psoriasis (n = 39) being the most frequent. Two patients had a diagnosis of connective tissue disease, presenting with back pain and generalised arthralgia respectively. In total, eight patients entered clinical trials following their first attendance in clinic. Two patients had been referred for a second clinical opinion; one for diagnostic and the other for treatment opinions. Further analysis, including a comparison of “choose and book” and triaged referrals, will be presented at the meeting.

## Discussion

The audit found that the majority of referrals to the Leeds SpA clinic are appropriate. Indeed, even among the patients with an eventual diagnosis of mechanical joint pain, a number had risk factors for spondyloarthritis and were reviewed in the appropriate setting. We can therefore say that presentation to this clinic is reasonably specific. However, the audit cannot assess the sensitivity of referral of SpA within the service. This would require an audit of all new referrals to the Leeds Rheumatology Service and may be an avenue of further investigation.
